# Physico-Chemical Investigation and Antimicrobial Efficacy of Ozonated Oils: The Case Study of Commercial Ozonated Olive and Sunflower Seed Refined Oils

**DOI:** 10.3390/molecules29030679

**Published:** 2024-02-01

**Authors:** Silvia Puxeddu, Alessandra Scano, Mariano Andrea Scorciapino, Ilenia Delogu, Sarah Vascellari, Guido Ennas, Aldo Manzin, Fabrizio Angius

**Affiliations:** 1 Department of Biomedical Sciences, Section of Microbiology and Virology, University of Cagliari, 09042 Cagliari, Italy; silvia.px@live.com (S.P.); deloguilenia@gmail.com (I.D.); svascellari@unica.it (S.V.); aldomanzin@unica.it (A.M.); 2 Department of Chemical and Geological Sciences, University of Cagliari, 09042 Cagliari, Italy; alessandra.scano@unica.it (A.S.); scorciapino@unica.it (M.A.S.); ennas@unica.it (G.E.); 3 Research Unit of the National Consortium of Materials Science and Technology (INSTM), University of Cagliari, 09042 Cagliari, Italy

**Keywords:** ozone, olive oil, sunflower oil, antimicrobial, ozonides

## Abstract

Drug resistance represents one of the great plagues of our time worldwide. This largely limits the treatment of common infections and requires the development of new antibiotics or other alternative approaches. Noteworthy, the indiscriminate use of antibiotics is mostly responsible for the selection of mutations that confer drug resistance to microbes. In this regard, recently, ozone has been raising interest for its unique biological properties when dissolved in natural oils. Ozonated oils have been reported to act in a non-specific way on microorganisms hindering the acquisition of advantageous mutations that result in resistance. Here, we focused on the antimicrobial effect of two commercial olive (OOO) and sunflower seeds (OSO) oils. Nuclear magnetic resonance spectroscopy and thermal analysis showed the change in the chemical composition of the oils after ozonation treatment. Different ozonated oil concentrations were then used to evaluate their antimicrobial profile against *Candida albicans*, *Enterococcus faecalis*, *Staphylococcus aureus*, *Klebsiella pneumoniae*, *Pseudomonas aeruginosa*, and *Escherichia coli* by agar diffusion and broth dilution methods. Cytotoxicity was also evaluated in keratinocytes and epithelial cells. Overall, our results revealed that both OOO and OSO showed a potent microbicidal effect, especially against *C. albicans* (IC50 = OOO: 0.3 mg/mL and OSO: 0.2 mg/mL) and *E. faecalis* (IC50 = OOO: 0.4 mg/mL and OSO: 2.8 mg/mL) albeit exerting a certain effect also against *S. aureus* and *E. coli.* Moreover, both OOO and OSO do not yield any relevant cytotoxic effect at the active concentrations in both cell lines. This indicates that the ozonated oils studied are not toxic for mammalian cells despite exerting a potent antimicrobial effect on specific microorganisms. Therefore, OOO and OSO may be considered to integrate standard therapies in the treatment of common infections, likely overcoming drug resistance issues.

## 1. Introduction

Infectious diseases are a significant global threat, as proved by the current emergence of highly transmissible pathogens such as SARS-CoV-2, liable for more than half a billion confirmed cases and more than 6 million deaths worldwide [1].

Although airborne transmission represents a highly effective route for the spread of a large number of pathogens behind contagious diseases (e.g., COVID-19, common colds, influenza, tuberculosis), indirect and vehicle transmission (i.e., via fomites and human interactions) depict a further crucial transmission way. Moreover, the emergence and spread of drug-resistant pathogens, which largely limit the treatment of common infections, have been calculated to likely cause more than 10 million deaths worldwide by 2050 if no new effective therapies are implemented [2]. Therefore, the development of new antibiotics or other alternatives is required as reported by WHO [3].

Noteworthy, bacteria belonging to the ESKAPEE group that exhibit multidrug resistance and virulence (*Enterococcus faecium*, *Staphylococcus aureus*, *Klebsiella pneumoniae*, *Acinetobacter baumannii*, *Pseudomonas aeruginosa*, *Enterobacter* spp., and *Escherichia coli*) are of particular importance. In fact, they are responsible for infections related to multi- and pan-drug resistance that are difficult or not treatable with existing antimicrobials, deserving the epithet “superbugs” [2].

In this context, personal hygiene measures are the first strategy against the spread of microorganisms within communities such as schools and hospitals. Among the various available options, the daily use of detergents and sanitizers is certainly the most important, which include alcohol or quaternary ammonium-based compounds. Despite the effectiveness, their use has been widely demonstrated to correlate with variable degrees of skin damage likely due to long-term use [4,5,6]. Recently, beyond synthetic and natural origin molecules studied to tackle microbial infections, ozone—a potent oxidant—is recently raising interest.

Ozone presents unique biological properties that justify its frequent use in removing chemical micro-pollutants [7] and inactivating bacteria through highly reactive radicals production [8,9,10,11,12]. Moreover, it can be applied in medical treatments (e.g., external ulcers, skin lesions, arterial circulatory disorders, and fungal infections) due to its antimicrobial [13,14,15,16,17,18,19], viral inactivation [20,21], antihypoxic, analgesic, and immunostimulating effects [22,23,24,25,26,27,28]. Nevertheless, ozone can be harmful when inhaled and should be used according to regulations and precautions to avoid inadvertent exposure [29]. Beyond that, it can be applied in different ways depending on the pathology to be treated [30]. It is rapidly perishable with a half-life of about 1 h at room temperature resulting in spontaneous oxygen gas formation [31,32]. It is difficult to use topically when diluted in water [33], but it can be dissolved in an oily medium, lending a wide range of antimicrobial activity to the oil-tackling microorganisms without causing cell or tissue damage.

In this scenario, our study aims to evaluate the specific antimicrobial efficacy of two different ozonated refined oils from olive and sunflower seeds, respectively. We tested them over a panel of opportunistic and pathogenic microbes among Gram-positive, Gram-negative, and fungi, typically present in the human skin and responsible for common cutaneous infections (e.g., cellulitis, erysipelas, impetigo, folliculitis, and candidiasis). Among the panel of microorganisms, catalase- and oxidase-positive microbes with great adaptability were also included. We focused our studies on evaluating the antimicrobial effect of the ozonated oil on these microorganisms and whether this effect may differ with the vegetable oil used. Cytotoxicity was also evaluated in keratinocytes (HaCaT) and epithelial (Vero) cells by viability assay.

## 2. Results

### 2.1. Thermogravimetric Analysis and Differential Scanning Calorimetry

**Figure 1:**
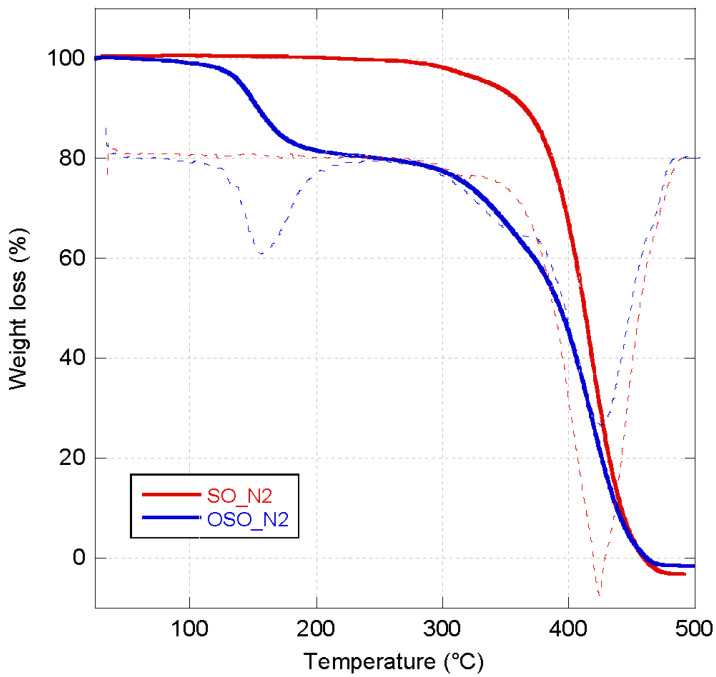
TGA thermograms (thick line) and related DTG (dotted line) of SO (red curve) and OSO (blue curve) measured under the N_2_ atmosphere.

**Figure 2:**
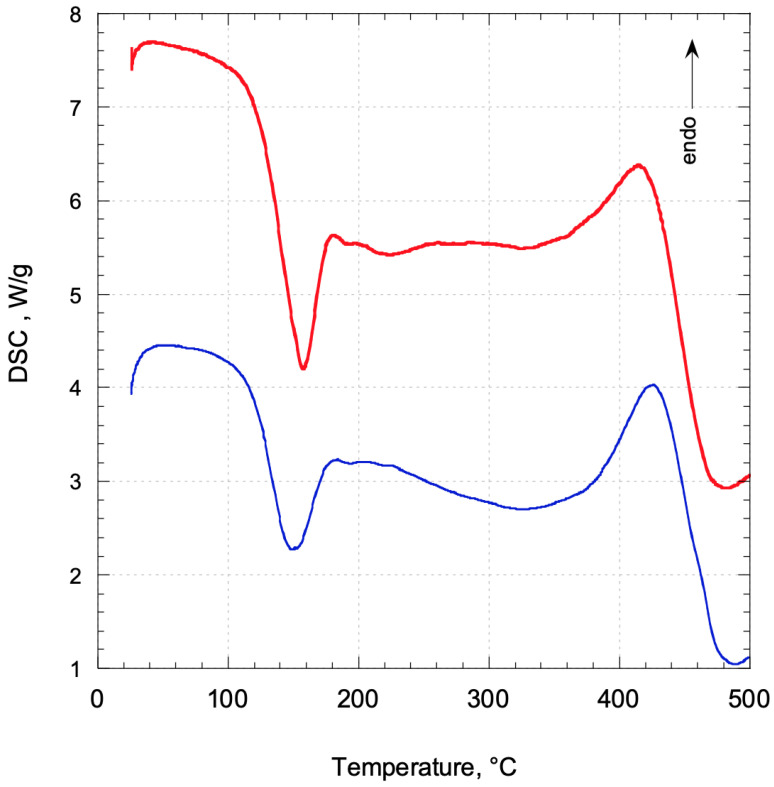
DSC thermograms of OOO (red curve) and OSO (blue curve).

**Figure 3:**
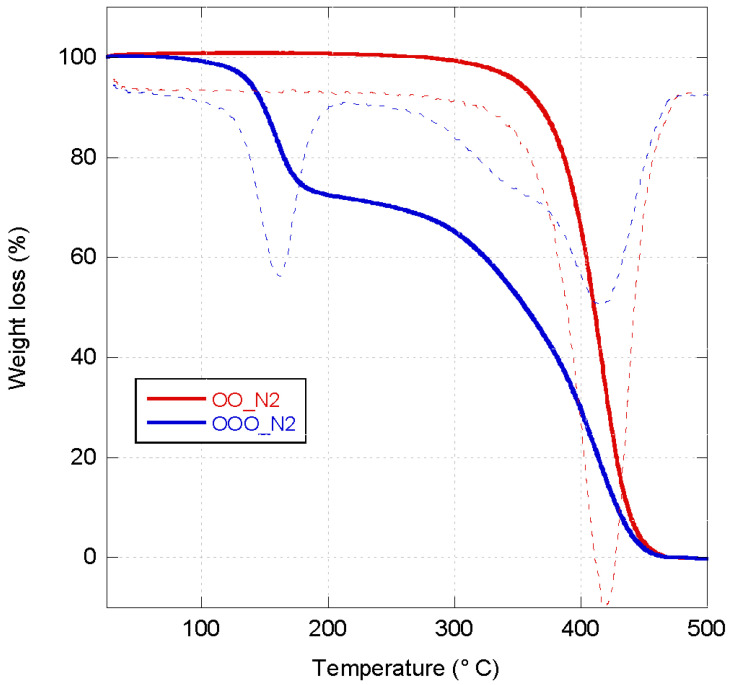
TGA thermograms (thick line) and related DTG (dotted line) of OO (red curve) and OOO (blue curve) measured under the N_2_ atmosphere.

TG curves in the N_2_ atmosphere for sunflower seed oil (SO) before and after being ozonated (OSO) are shown in Figure 1. SO showed only a mass loss in the range between 300 and 480 °C, while OSO presented two mass losses, as confirmed by the related dTG curves. The first mass loss is visible in the temperature range of 100–200 °C related to the decomposition of ozonides obtained after the ozonation of SO, while a second one is observed between 300 and 480 °C due to the complete decomposition of SO. TG results are confirmed by DSC thermograms of OSO that show an exothermic peak centered at 157 °C typical of ozonated oils (Figure 2) [34]. It is attributed to degradation followed by decomposition of ozonides, with a thermal decomposition enthalpy (ΔH) of −306 J/g. A similar behavior was also observed for the olive oil (OO) and ozonated olive oil (OOO). In fact, while only a mass loss in the range between 300 and 500 °C was visible in the thermograph of OO, two mass losses in the ranges of 100–200 °C and 300–500 °C, respectively, were evident for the OOO (Figure 3). The DSC thermogram of OOO showed an exothermic peak centered at 151 °C (Figure 2) with a thermal decomposition enthalpy (ΔH) of −361 J/g.

### 2.2. Nuclear Magnetic Resonance Spectroscopy

**Figure 4:**
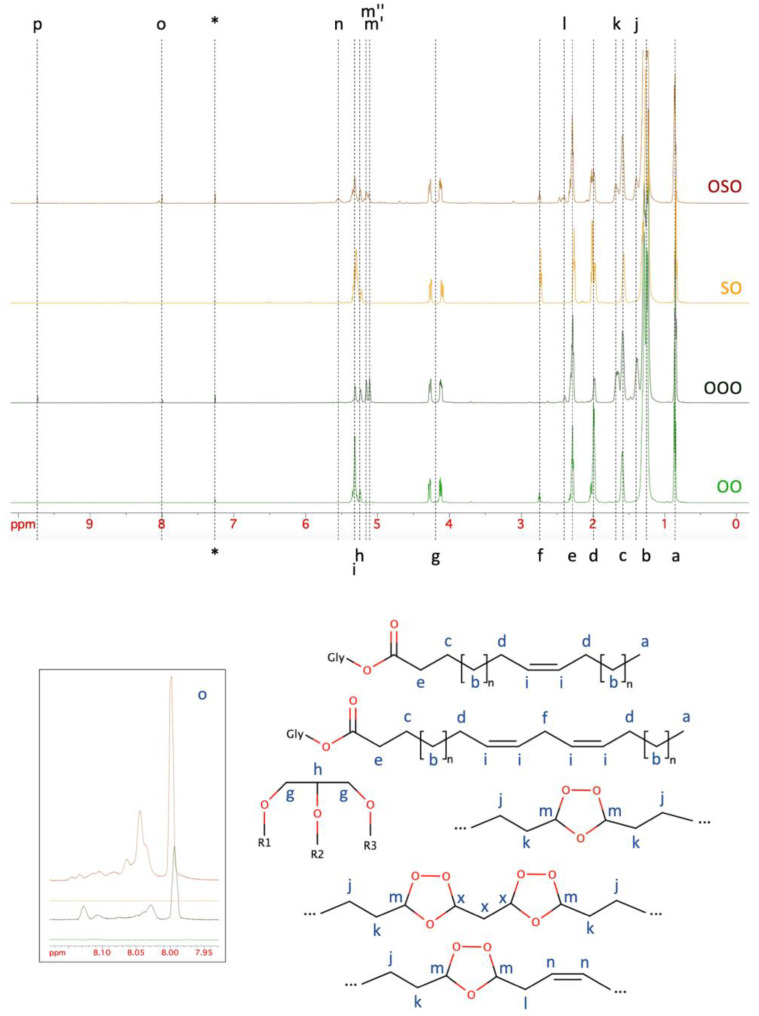
The H-NMR stack plot of the refined olive oil (OO) and sunflower seed oil (SO) and the corresponding ozonated oils, OOO and OSO, respectively. Lowercase letters are used to label the main resonances in the spectra. The ones already present in the refined oils are reported along the bottom side, while those that appeared after ozonation are reported along the top side of the plot. The asterisk is used to label the residual CHCl_3_ resonance. The region around 8 ppm is expanded in the left bottom corner of the figure. The Lewis structures (H atoms are not shown for clarity) of the typical vegetable oil components and the identified ozonation products are shown together with lowercase letters to indicate resonance assignments. The label “x” is used to indicate missing (or very low) resonances.

The stack plot of the ^1^H spectra of the two refined oils, namely, olive and sunflower seeds, before and after ozonation is shown in Figure 4, together with the Lewis structure of the typical oil components and their ozonation products. The intensity of the resonances d, f, and i, which are characteristics of fatty acid unsaturations, was dramatically decreased by ozonation. In fact, a number of new resonances of significant intensity appeared in the two spectra after ozonation. In particular, based on literature data [34], resonance j could be assigned to the β-methylene groups of newly formed 1,2,4-trioxolane rings. The resonance k could be assigned to the α-methylene groups of a single 1,2,4-trioxolane ring, while it is worth noting that α-methylene groups between two consecutive oxolane rings were expected at higher frequency (2.0–2.1 ppm). Only in the case of OSO, an extremely low signal was observed and possibly attributed to the latter. Conversely, the resonance l was quite visible in the spectrum of both OOO and OSO, which was assigned to the single methylene group separating one 1,2,4-trioxolane ring and one unreacted double bond. These observations indicated that the reaction of ozone with polyunsaturated fatty acid rarely produced more than a single oxolane ring per fatty acid chain. This was bolstered by the absence of the resonance due to oxolane methine protons located on the side of one adjacent oxolane ring (ca. 5.40 ppm). In fact, resonance n was quite visible in the spectrum of OSO, and this was attributable to the olefinic protons next to one 1,2,4-trioxolane ring. Finally, the two resonances m′ and m″ are due to all the oxolane methine protons but those located on the side of adjacent oxolane rings. The products of the ozonation reported in Figure 4 are due to the formation of a primary 1,2,3-trioxolane, which spontaneously decomposes into a carbonyl oxide and a carbonyl compound that finally react to form the 1,2,4-trioxolane ring [35]. However, it is also possible that the two intermediates are further degraded to formaldehyde (SDBS n. 9410; https://sdbs.db.aist.go.jp/sdbs/cgi-bin/cre_index.cgi (accessed on 20 October 2023) and formic acid (SDBS n. 10523), whose resonances are clearly visible in Figure 4 with label p and o, respectively. By expanding the region of the latter, especially in the case of OSO, other resonances could be observed, which are possibly attributed to different formates. Integration of the resonances allowed us also to retrieve quantitative information to compare the two ozonated oils in detail. Table 1 presents the results. This semi-quantitative information bolstered the above-mentioned differences between the two oils. The triacylglycerols appeared to be intact, with 3 chains per glycerol unit, either before or after ozonation. The average number of unsaturations was larger in the SO than in OO and decreased dramatically due to ozonation by about the same extent. Polyunsaturations were more abundant in SO than in OO. After ozonation, the number of chains with one 1,2,4-trioxolane ring was slightly larger for OOO than OSO, and this was reflected by the significantly larger number of formates found in the OSO with respect to OOO. Moreover, this can explain the slightly higher ΔH value of OOO compared to OSO obtained by the DSC analysis. The amount of formaldehyde per glycerol was virtually the same.

### 2.3. Cytotoxicity

**Figure 5:**
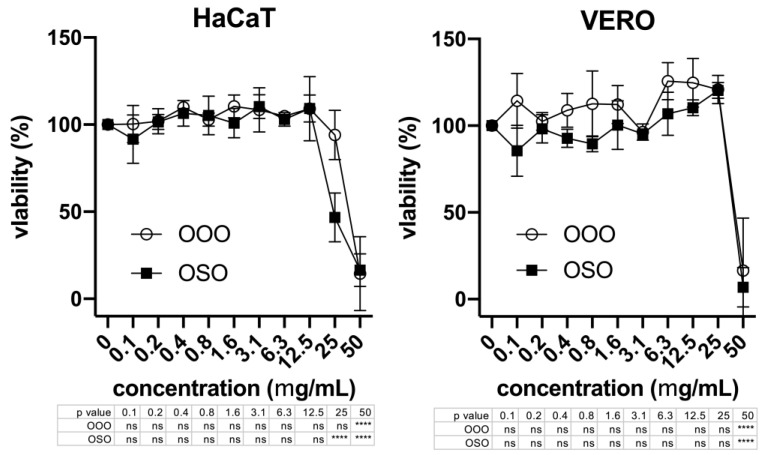
Cell viability of keratinocytes (HaCat) and epithelial (Vero) cells after 24 h treatment with ozonated olive (OOO) and sunflower (OSO) oils assessed by MTT assay. Data are expressed as the means ± standard errors in the percentage of the untreated control as a function of the concentration (x axis). Below each graph, the *p* values are reported vs. untreated control as calculated by 2-way ANOVA and Fisher’s LSD test. **** *p* < 0.0001; ns, not significant.

Over a 24 h treatment, OOO and OSO do not exert any cytotoxic effect at the active concentrations in both cell lines tested, as obtained by MTT assay. Nevertheless, high concentrations of OOO (CC50 = 48 mg/mL) and OSO (CC50 = 46 mg/mL) showed to be toxic for Vero cells. On the other hand, HaCaT cells were shown to be slightly more susceptible to OSO (CC50 = 28 mg/mL) than OOO (CC50 = 40 mg/mL) (Figure 5 and Table 2).

### 2.4. Antimicrobial Effect

**Figure 6:**
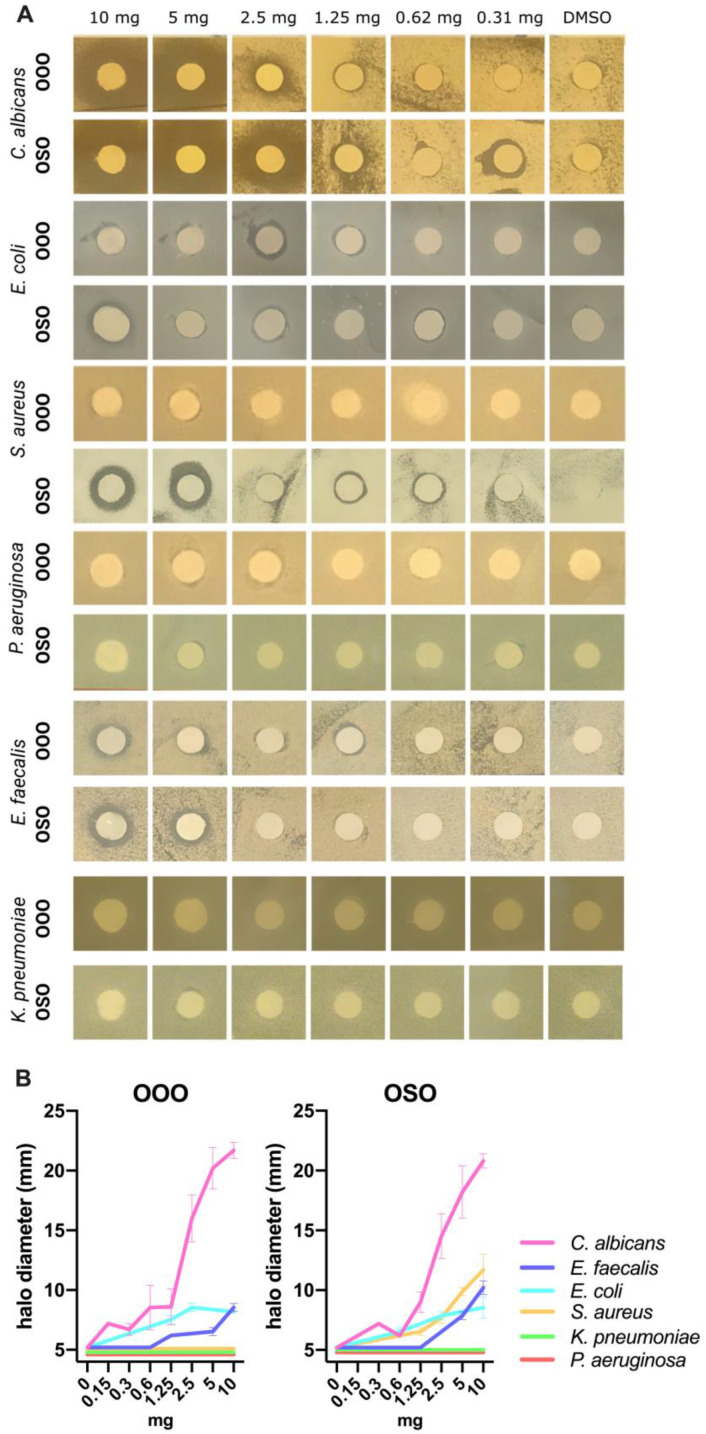
Susceptibility profile to OOO and OSO against all the microbes assessed by Kirby Bauer agar diffusion method (**A**). Different amounts (10, 5, 2.5, 1.25, 0.62, and 0.31 mg) of ozonated oils were reported together with the negative control (DMSO) and the diameters of the inhibition halos were expressed as the means ± standard errors (**B**).

**Figure 7:**
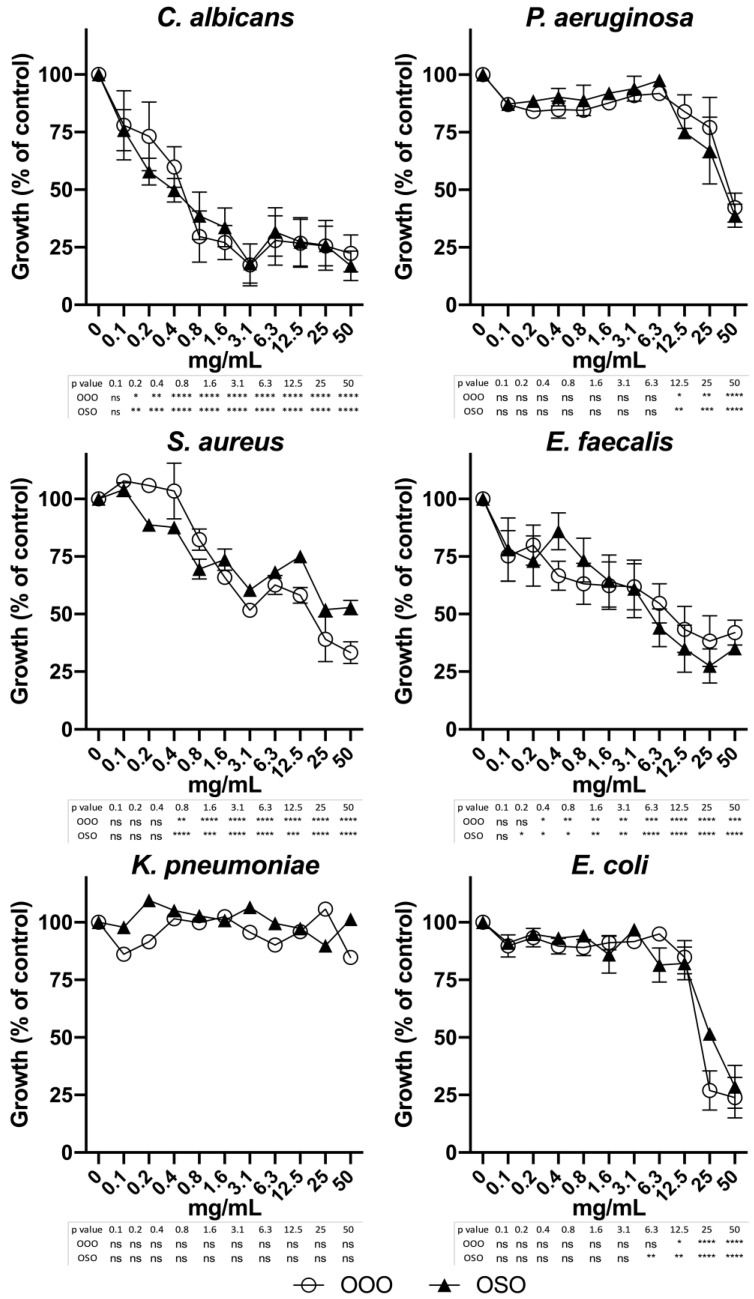
Bactericidal effect after 24 h treatment with ozonated olive (OOO) and sunflower (OSO) oils assessed by microdilution assay. Data are expressed as the means ± standard errors in the percentage of the control’s growth as a function of the concentration (x axis). Below each graph, the *p* values are reported vs. untreated control as calculated by 2-way ANOVA and Fisher’s LSD test. * *p* < 0.05; ** *p* < 0.01; *** *p* < 0.001; **** *p* < 0.0001; ns, not significant.

Preliminary experiments with non-ozonated refined oils (SO and OO) revealed the absence of any antimicrobial activity accounting for its assessment exerted by the ozonated ones. As shown in Figure 6, the agar diffusion test revealed that *C. albicans* and *E. faecalis* were the most susceptible to ozonated oils, among the panel of microbes we tested. In particular, the most evident inhibition was observed for *C. albicans*, even at low concentrations with the best results obtained with OOO (0.15 mg). Analogously, *E. faecalis* was shown to be more inhibited by OSO than OOO (1.25 vs. 5 mg). Interestingly, *S. aureus* showed a sharp susceptibility to OSO (0.6 mg), whereas it appeared resistant to OOO. In addition, also *E. coli* showed inhibition halos, albeit quickly reducing its activity by dilution, starting from 10 to 2.5 mg of OSO and OOO. Finally, *K. pneumoniae* and *P. aeruginosa* did not show any susceptibility profile to ozonated oils by the agar diffusion assay (Figure 6). Moreover, regarding the MIC determination by broth microdilution method, we observed high interference of the oils with the optical density in the absence of microbe in particular at the concentration range of 3.1–25 mg/mL, resulting in the impossibility of evaluating the MIC (Figure S1). Therefore, we rather opted to evaluate the bactericidal activity obtaining more reliable and repeatable data, and the IC50 for each ozonated oil was determined. Although both OOO and OSO did not show relevant inhibitory effect against *P. aeruginosa* (OOO: >50 mg/mL; OSO: 41.2 mg/mL) and *K. pneumoniae* (OOO: >50 mg/mL; OSO: >50 mg/mL) at any tested concentrations as expected, a bactericidal activity was observed against *E. coli* (OOO: 16.7 mg/mL; OSO: 27.5 mg/mL) and *S. aureus* (OOO: 10.7 mg/mL; OSO: 24.7 mg/mL). Interestingly, the most outperforming results were obtained for both ozonated oils against *C. albicans* (OOO: 0.3 mg/mL; OSO: 0.2 mg/mL) and *E. faecalis* (OOO: 0.4 mg/mL; OSO: 2.8 mg/mL). However, significant growth inhibition was observed for concentrations lower than the IC50 values (Table 2 and Figure 7) with the lowest active concentration equal to 0.2 mg/mL for both oils against *C. albicans*, 0.4 (OOO) and 0.2 (OSO) mg/mL against *E. faecalis*, 0.8 mg/mL for both against *S. aureus*, 12.5 (OOO) and 6.3 (OSO) mg/mL against *E. coli*, and 12.5 mg/mL for both oils against *P. aeruginosa* (Figure 7).

Overall, our results suggest that ozonated oils exert antimicrobial activity against common bacteria and fungi responsible for major skin and nosocomial infections with negligible toxicity for mammalian cells at effective doses. In particular, our results indicate both ozonated oils to be very promising and safe molecules against *C. albicans* showing a very high selectivity index (SI) equal to 151 (OOO) and 145 (OSO) in keratinocytes, 182 (OOO) and 242 (OSO) in epithelial cells. Interestingly, the SI related to *E. faecalis* was far higher for OOO (114 in HaCat and 137 in Vero) than for OSO (10 in HaCat and 17 in Vero). On the other hand, relevant effects against *P. aeruginosa* (OSO: 1) and *K. pneumoniae* were not observed; however, SI was obtained for both *E. coli* in HaCat (OOO: 2 and OSO: 1) and Vero (OOO: 3 and OSO: 2), as well as for *S. aureus* in HaCat (OOO: 4 and OSO: 1) and in Vero (OOO: 5 and OSO: 2), albeit much lower compared to *C. albicans* and *E. faecalis* (Table 2).

## 3. Discussion

Sunflower seed and olive oils are two common products widely used in the culinary, cosmetic, and pharmaceutical fields, obtained from the processing of sunflower seeds and pressing of olives, respectively. They mostly differ in the fatty acid composition. Sunflower seed oil presents a high portion of the polyunsaturated fat linoleic acid (48–74%) and less content of the monounsaturated fat oleic acid (14–39%), while olive oil contains a high oleic acid amount (65–85%) [36].

Recently, they have been chosen as substrates for the ozonation process after a refining of the raw oils, to obtain highly effective antimicrobial products, due to the formation of oxygenated compounds [34,37,38,39]. Refining is a necessary treatment of these vegetable oils that will be used in culinary, cosmetic, and pharmaceutical applications. It consists of removing free fatty acids, unsaponifiable matters, waxes, pigments, solid impurities or pollution from storage, transport, and processing of the oil, which have a negative impact on the oil quality and stability [40].

During the ozonation process of the refined oils, the reaction of ozone with the carbon–carbon double bonds of the unsaturated fatty acids occurs, giving rise to the formation of ozonides, different types of peroxide species, and aldehydes, responsible for the wide antimicrobial activity of the ozonated oils [34,38]. We selected commercially refined sunflower seed (SO) and olive (OO) oils to study their antimicrobial activity after ozonation. This process yields two products, namely, OSO and OOO, both showing a change in color as well as an increased viscosity of 180 and 86 mPa·s, respectively (ozonated oil technical sheets, supporting information).

The successful ozonation of the two refined oils was first shown by the analytical studies performed by the vendor, which showed an important increase in the oxidation number (meqO2/Kg) of the ozonated oils as reported in Table 3. Thermal analysis and the NMR characterization further confirmed the change in the chemical composition of SO and OO after ozonation treatment. All the resonances due to the triacylglycerols expected for vegetable oils were identified. The resonance assignment and their relative intensities confirmed that monounsaturated fatty acids were the major fraction of OO, while polyunsaturated chains were much more abundant in the SO, in agreement with the data sheets provided by the vendor (ozonated oil technical sheets, supporting information).

After ozonation, the intensity of the resonance characteristics of fatty acid unsaturations dramatically decreased. This suggests that ozone was not merely present in the oil in the form of microbubbles, but reacted with the fatty acid chain unsaturations, giving rise to the formation of the chemical species likely responsible for the antimicrobial activity.

Ozonated oils have been studied in vivo and in vitro for their antimicrobial activity with promising results in inhibiting the growth of bacteria and fungi, suggesting their potential use as antimicrobial agents particularly interesting for topical applications. In fact, several in vivo studies have demonstrated their efficacy in healing severe skin lesions caused by *S. aureus* and MRSA, with limited side effects and costs [41]. Sechi et al. investigated the antimicrobial activity of ozonated sunflower oil against various pathogens including *S. aureus*, *E. faecalis*, *E. faecium*, *S. pyogenes*, *E. coli*, *P. aeruginosa*, and different species of *Mycobacterium*, reporting a valuable activity against all the microorganisms tested [39,41,42]. This antibacterial activity has been confirmed by Serio et al. in both Gram-negative and Gram-positive bacteria, as well as against parasites [41,42].

Additionally, in vitro studies have shown a potent bactericidal effect of ozonated oils against cutaneous infections determining a sharp reduction in the bacterial load of methicillin-sensitive *S. aureus* and MRSA [42,43]. Therefore, further research into the characteristics of ozonated oil and its ozonation process may optimize and improve antimicrobial therapy, potentially offering a valuable treatment option in dermatology and other medical fields.

Here, we focused on evaluating the antimicrobial effect of two different types of refined ozonated oils over a panel of opportunistic and pathogenic microbes that cause common skin infections, namely, *C. albicans*, *E. faecalis*, *S. aureus*, *K. pneumoniae*, *P. aeruginosa*, and *E. coli*. Differently from other studies, in our experimental conditions, OOO and OSO showed a potent microbicidal effect, especially against *C. albicans* and *E. faecalis*. However, although no relevant effect was observed against *K. pneumoniae* and *P. aeruginosa*, our results are in line with previous studies showing some susceptibility of *E. coli* and *S. aureus* when challenged with ozonated oils.

In addition, to verify how effective and safe OOO and OSO treatment would be in vivo, we calculated the selectivity index (SI) by the ratio of the cytotoxic concentration (CC50) in keratinocytes and epithelial cells over the inhibitory concentration (IC50) (Table 2). Overall, our results indicate that these ozonated oils are not toxic for mammalian cells and exert potent antimicrobial effects on specific microorganisms with significant activity exerted by concentrations lower than the IC50 values (Figure 7). In particular, our results indicate both OOO (SI: 151–182) and OSO (SI: 145–242) to be very promising against *C. albicans*. Noteworthy, the SI was far higher for OOO (SI: 114–137) than OSO (SI: 10–17) against *E. faecalis*. SI was also obtained for both *E. coli* (OOO: 2–3; OSO: 1–2) and *S. aureus* (OOO: 4–5; OSO: 1–2) albeit much lower compared to *C. albicans* and *E. faecalis* (Table 2).

These results reinforce previous findings suggesting that they may be considered to complement standard therapies in the treatment of common infections (e.g., mycosis). In this context, one of the most used topical therapies in the treatment of common skin infections is gentamicin-based with cortisone, which implicates its use exclusively in the absence of ulcers and/or lesions. Conversely, ozonized oils have indeed been widely reported to favor wound healing, suggesting and supporting their use also in these cases. Furthermore, it is plausible that ozonated oils should be able to overcome drug resistance issues due to the non-specific mechanisms of action of the ozone. In fact, while drug resistance arises by the selection of microbe strains capable of inactivating a drug (i.e., β-lactamases), modifying molecular targets (i.e., mutations conferring less affinity, increasing the expression), or even increasing the efflux pump activity, the ozonated oils induce significant cell wall and cytoplasmic membrane alterations resulting in cell death. This mechanism is totally not specific and may explain their wide-spectrum antimicrobial activity. In fact, it was already suggested that ozonated oils’ activity was related—yet to be clearly identified—to oxidizing species rather than to the mere peroxide index [35], being active also against multidrug-resistant microbes. These observations support the hypothesis that these kinds of compounds are immune from the emergence of resistance mechanisms.

**Table 3:** Physico-chemical characteristics * of ozonated olive and sunflower oils.

	Ozonated Olive Oil	Ozonated Sunflower Oil
Number of oxidation (meqO_2_/kg)	3110 (2900–3300)	3520 (3000–3600)
Viscosity (mPas)	86 (80–200)	180 (80–250)
Acidity (mg KOH/g)	9.93	28
Turbidity color (NTU/FTR)	<1 (0 ÷ 20)	<1 (0–20)
Density 20 °C (g/cm^3^)	0.900 (0.800–1000)	0.990 (0.920–1.000)

* Data provided by the vendor (OS Srl, IT) in respect of the guidelines from the International Scientific Committee of Ozone Therapy (ISCO3) [44,45,46].

## 4. Materials and Methods

### 4.1. Materials

Ozonated olive oil (Pure Oil 100% olio di oliva ozonizzato) and ozonated sunflower oil (Pure Sun 100% ozonized sunflower oil) were kindly provided by OS Srl (Pesaro, Italy). The respective physico-chemical characteristics of the ozonated oils are reported in Table 3 (ozonated oil technical sheets, supporting information). Chloroform-d (99.8 atom % D, 0.03 % (*v*/*v*) TMS) for the nuclear magnetic resonance spectroscopy was purchased from Sigma-Aldrich (St. Louis, MO, USA).

### 4.2. Physico-Chemical Characterization of Ozonated Oils

#### 4.2.1. Thermal Characterization and Differential Scanning Calorimetry

A PerkinElmer (Waltham, MA, USA) STA6000 instrument was used to carry out simultaneous thermogravimetric (TGA) and differential scanning calorimetry (DSC) analysis under a nitrogen atmosphere (40 mL min^−1^). Four milligrams of the sample were placed in an alumina crucible using a micropipette, and measurements were performed in the temperature range of 25−500 °C (heating rate: 10 °C min^−1^; temperature accuracy: ±1 °C). Each analysis was carried out in triplicate.

#### 4.2.2. Nuclear Magnetic Resonance Spectroscopy (NMR)

^1^H-NMR spectra were recorded at 27 °C on a Bruker Avance III HD 600 (Bruker, Billerica, MA, USA) operating at a ^1^H frequency of 600.134 MHz. A few drops of the oil were inserted in a 5 mm NMR tube and dispersed with 600 μL of CDCl_3_. The samples were always protected from light by wrapping the vials and NMR tubes with aluminum film before inserting the tube into the spectrometer. ^1^H spectra were acquired with 8 transients, 3.17 μs (30°) hard pulse, 40.0 s relaxation delay, 2.7 s acquisition time, and a spectral width of 12.019 kHz. The residual CHCl_3_ resonance was used to calibrate the ppm scale (7.26 ppm from trimethylsilane, iNMR software V6.4.5, Mestrelab Research S. L., Santiago, Spain).

### 4.3. Cell Viability Assay

Immortalized human keratinocytes (HaCaT) and African green monkey kidney (Vero) cells (ATCC collection, Manassas, VA, USA) were grown in Dulbecco’s modified Eagle’s medium (DMEM; Corning Inc., Corning, NY, USA) supplemented with 10% fetal bovine serum (FBS), 1% penicillin/streptomycin and incubated at 37 °C with 100% humidity and 5% CO_2_. Cells were seeded in 96-well plates at a density of 7.5 × 10^3^ cells/well and after 24 h of incubation, cells were exposed for 24 h to ozonated oils at different concentrations (from 50 to 0.1 mg/mL) that were inspired by other studies [37]. Cell viability was assessed by adding 450 μM MTT (3(4,5-dimethylthiazolyl-2)-2,5-diphenyltetrazolium bromide) (Sigma Aldrich, Saint Louis, MO, USA) to each well [47]. After 2–3 h, the formazan crystals were dissolved in DMSO and the solution absorption was measured at 570 nm with a microplate reader (Tecan Infinite 200, Männedorf, Switzerland). All the experiments were repeated at least three times and in triplicate. The results are presented as the percent of cell viability of untreated cells (control).

### 4.4. Microbial Strains and Culture Conditions

The bacterial and fungal strains were purchased from ATCC. *Staphylococcus aureus* (ATCC 25923) and *Pseudomonas aeruginosa* (ATCC 27853) were cultured in Tryptic Soy Broth (TSB), *Escherichia coli* (ATCC 25922) was cultured in Lennox Broth (LB), *Enterococcus faecalis* (ATCC 29212) and *Klebsiella pneumoniae* (ATCC 13883) were cultured in Nutrient Broth (NB), and *Candida albicans* (ATCC 10231) in Sabouraud Broth (SB). All the culture media were purchased from Condalab Italia. The stock cultures were stored at −80 °C in the appropriate broth with 20% glycerol. For each strain, a growth curve was preliminarily performed, and the standard suspension was determined (5 × 10^7^ CFU/mL for all bacteria and 5 × 10^6^ CFU/mL for *C. albicans*) to minimize the variation in experimental conditions and ensure reproducible experimental data. During the experiments, the strains were incubated at 37 °C with shaking (200 rpm) for 24 h (48 h for *K. pneumoniae* and *E. faecalis*).

### 4.5. Antimicrobial Activity

Microorganisms were challenged with different ozonated oils concentrations obtained by two-fold serial dilutions in dimethyl sulfoxide (DMSO; Sigma Aldrich, Saint Louis, MO, USA), which was used as a negative control, to evaluate the antimicrobial resistance/susceptibility by agar diffusion [48] and broth microdilution methods.

#### 4.5.1. Agar Diffusion Test (Kirby–Bauer)

For each strain, 15 mL of Mueller Hinton (MH) agarized medium (Sabouraud Agar for *C. albicans*) heated up at 55 °C was added to a 90 mm Petri dish, and after agar solidification, 50 μL from the standardized suspensions of each strain was inoculated onto the agar plate using a sterile swab. Blank sterile paper disks (5 mm in diameter) were laid on each Petri dish [49] and soaked with 20 μL of the ozonated oil samples, resulting in final concentrations ranging from 10 to 0.04 mg. After 24–48 h of incubation at 37 °C, plates were checked and photographed and the inhibitory activity was recorded by the presence of the inhibition halo.

#### 4.5.2. Broth Dilution Tests

Minimum inhibitory concentration (MIC) and minimum bactericidal concentration (MBC) were assessed in sterile 96-well plates (Thermo Fisher Scientific, Norristown, OA, USA) by the micro-dilution method following CLSI procedures [50]. Each well was poured with 200 μL of standardized microbial suspension and 5% (*v*/*v*) of different concentrations of ozonated oil samples reaching the final concentrations ranging from 50 to 0.1 mg/mL, in line with other studies [37] and guided by the viability assay results. Untreated microbes were the control group. The cultures were incubated at 37 °C for 24–48 h and OD600 was measured with a microplate reader (Tecan Infinite 200, Switzerland). After that, 10 μL collected from each well was seeded in a new plate with fresh medium (200 μL/well) to determine the lowest concentration that demonstrates a consistent growth reduction (MBC) after 24–48 h of incubation when compared to untreated control assessed by OD600 with a microplate reader (Tecan Infinite 200, Männedorf, Switzerland).

### 4.6. Statistics

Statistical analysis was performed with GraphPad Prism version 8.2.1 (GraphPad Software, San Diego, CA, USA) for macOS [51]. All data were expressed as the means ± standard errors from at least three independent experiments in triplicate and analyzed by the unpaired Student’s *t*-test, Mann–Whitney U test, or 2-way analysis of variance (ANOVA) followed by Fisher’s LSD test. Data were considered significant when *p* < 0.05.

## 5. Conclusions

Our study investigated the physico-chemical properties and antimicrobial activity of two commercial ozonated sunflower and olive oils, against *C. albicans*, *E. faecalis*, *S. aureus*, *K. pneumoniae*, *P. aeruginosa*, and *E. coli*. Both ozonated oils exhibited high microbicidal effects against *C. albicans* and *E. faecalis*, while they were active also against *S. aureus* and *E. coli.* Moreover, the cell viability assay showed no cytotoxicity in keratinocytes and epithelial cells. These results make the two oils studied promising for a wide range of applications in medicine and healthcare such as treatment of common infections, likely overcoming drug resistance issues. The treatment of vegetable oils with ozone creates a reservoir of antimicrobial species active against diverse microorganisms. In this scenario, further studies will be run on the delivery of the two ozonated oils by different pharmaceutical formulations.

## Figures and Tables

**Table 1:** The ratio between the integral of selected 1H NMR resonances.

Sample	OO	OOO	SO	OSO
^a^ Fatty acid/glycerol	3.21	3.10	2.89	3.01
^b^ Unsaturations/glycerol	3.06	0.57	4.03	0.92
^c^ Polyunsaturations/glycerol	0.32	0.00	1.53	0.31
^d^ Ozonated chains/glycerol	0.00	1.28	0.00	0.85
^e^ Formaldehyde/glycerol	0.00	0.10	0.00	0.09
^f^ Formates/glycerol	0.00	0.15	0.00	0.40

All the following integral values were divided by the integral of resonance h: ^a^ (⅓ resonance a); ^b^ (½ resonance i); ^c^ (½ resonance f); ^d^ (½ resonance m); ^e^ (½ resonance p); ^f^ (all the resonances in the range 7.95–8.15 ppm).

**Table 2:** Cytotoxic activity (CC), inhibitory concentration (IC), and selectivity index (SI) detection of ozonated oils after treatment.

CC50 (mg/mL)		IC50 (mg/mL)					
		*C. albicans*	*E. faecalis*	*E. coli*	*S. aureus*	*P. aeruginosa*	*K. pneumoniae*
OOO		0.3	0.4	16.7	10.7	>50	>50
HaCaT	40.1	*151*	*114*	*2*	*4*	*-*	*-*
Vero	48.2	*182*	*137*	*3*	*5*	*-*	*-*
OSO		0.2	2.8	27.5	24.7	41.2	>50
HaCaT	28.1	*145*	*10*	*1*	*1*	*1*	*-*
Vero	46.8	*242*	*17*	*2*	*2*	*1*	*-*

Abbreviations: HaCaT, immortalized human keratinocytes; Vero, African green monkey kidney cells; OOO, ozonated olive oil; OSO, ozonated sunflower seeds oil. Data are expressed as the mean of three independent experiments. CC50, a cytotoxic concentration that inhibits cell viability by 50%; IC50, an inhibitory concentration that inhibits microorganism growth by 50% detected by microdilution assay; SI, selectivity index (CC50/IC50) is reported in italics.

## Data Availability

The data presented in this study are available in the article and Supplementary Materials.

## Supplementary Materials

The following supporting information can be downloaded at: https://www.mdpi.com/article/10.3390/molecules29030679/s1, Figure S1: Different microbial culture broths were measured by optical density at 600 nm (OD600) in absence or presence of ozonated oils at 2-fold scalar concentrations. Data sheet: ozonated sunflower oil technical sheet and ozonated oilve oil technical sheet—reproduced with permission from the vendor.
