# Supplementary material for: Physico-Chemical Investigation and Antimicrobial Efficacy of Ozonated Oils: The Case Study of Commercial Ozonated Olive and Sunflower Seed Refined Oils
Source: Molecules. 2024 Feb 1;29(3):679. doi: 10.3390/molecules29030679 (PMC10856119; doi:10.3390/molecules29030679)
Supplement: Supplementary file 1 [file molecules-29-00679-s001.zip › Figure S1.pdf]

Supplementary Information

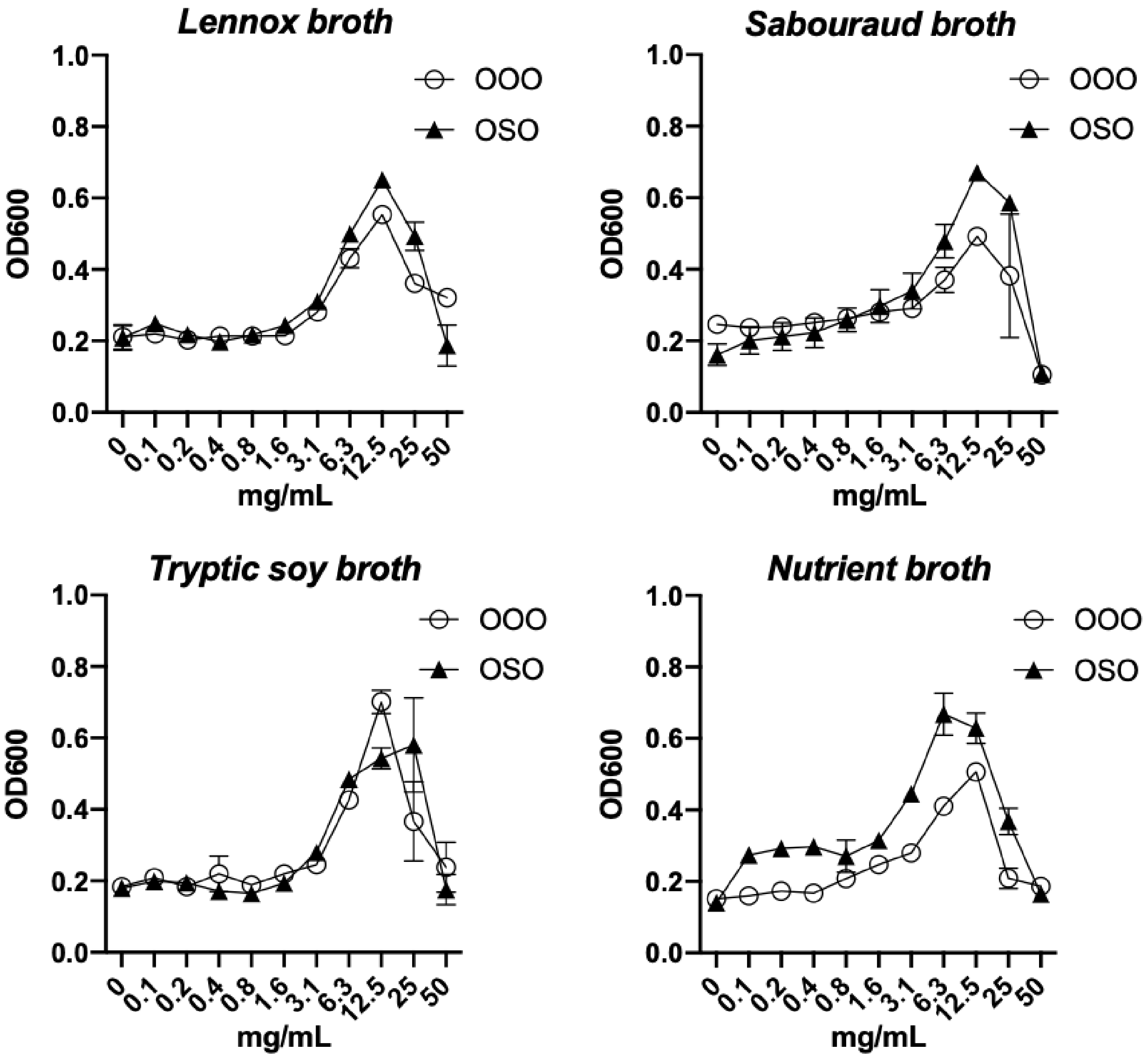

Figure S1. Different microbial culture broths were measured by optical density at 600 nm (OD600) in absence or presence of ozonated oils at 2-fold scalar concentrations.
