# Supplementary material for: Physico-Chemical Investigation and Antimicrobial Efficacy of Ozonated Oils: The Case Study of Commercial Ozonated Olive and Sunflower Seed Refined Oils
Source: Molecules. 2024 Feb 1;29(3):679. doi: 10.3390/molecules29030679 (PMC10856119; doi:10.3390/molecules29030679)
Supplement: Supplementary file 1 [file molecules-29-00679-s001.zip › ozonated olive oil technical sheet.pdf]

CONSORZIO FUTURO IN RICERCA

in collaborazione con Prof. P. G. Balboni
docente incaricato di insegnamento (MED/07) *come cultore della materia "Microbiologia" - Sezione di Microbiologia Università degli studi di Ferrara*

SCHEDA TECNICA

**Committente / *Customer*:**<br><br>**OS s.r.l.**<br>Viale Fratelli Rosselli, 46 – 61121 PESARO (PU) - Italia	Data ricevimento /<br>*Date of the sample:* 03/07/2018<br><br>*Data inizio analisi /*<br>*Start date analisys:* 05/07/2018<br><br>*Data fine analisi /*<br>*End date analisys:* 16/07/2018<br>Revisione:27/09/2021
**CODICE analisi /** *Analisys CODE N°:* 1807031	
**Prodotto / Sample description**	
**PURE OIL 100% ozonized olive oil**	**LOTTO N° 17418**

Nome prodotto	Pure oil 100% olio di oliva ozonizzato<br>Ozonized Olea europea (olive) fruit with Ozone
INCI NAME/IUPAC	OZONIZED OLEA EUROPEA (OLIVE) FRUIT OIL
Composizione	Olio di oliva ozonizzato (Olea europea oil, ozone)
EINECS	919-979-9
Descrizione	Prodotto per uso esterno a base di olio di oliva sottoposto ad ozonizzazione.
Categoria	Oli naturali
Aspetto a 20°C	Liquido oleoso traslucido, leggermente opalescente
Colore	Giallo chiaro paglierino
Odore	Profumazione caratteristica

PROVA ANALITICA CHIMICO FISICA	RISULTATO	Intervallo di accettabilità
pH = -log[H+] a 20°C	n.a.	non applicabile
Densità (g/cm^3) a 20°C	0,900	Conforme (limiti: 0.800 ÷ 1.000)
Viscosità (mPas)<br>Gir.5 rpm 20 a 25°C	86,0	Conforme (limiti: 80 ÷ 200)
Torbidità colore (NTU/FTU)	<1	Conforme (limiti: 0 ÷ 20)
Acidità (g ac. oleico/100 g)	9,93	Conforme (limite: <30)
Numero di ossidazione (meqO_2/kg)<br>(metodo ISCO3)	3110	Conforme (limiti: 2900 ÷ 3300)
Nichel test (mg/Kg)<br>APAT CNR IRSA 3220 Man29 2003	0.05	Conforme (limite quantitativo di prova di 0.05)
Solubilità	Non solubile in acqua, solubile in glicerina /paraffina Liposolubile in oli e disperdibile in emulsioni.	
Conservazione	Conservare in luogo fresco, asciutto e al buio; lontano da fonti di calore; accuratamente chiuso senza contatto con l'ambiente esterno.	
Settori di utilizzo	Cosmetico, farmaceutico uso topico, veterinario.	
Concentrazione di utilizzo	Da 0,1% al 100% (in funzione della formulazione)	

I risultati analitici si intendono riferiti esclusivamente al campione analizzato. Il presente Documento non può essere riprodotto neppure in forma parziale salvo approvazione scritta da parte del Responsabile. Questo report è valido elettronicamente, perché costituisce copia esatta controllata e firmata del certificato di analisi originale, conservato in accordo alle procedure di Norme di Buona Prassi di Laboratorio. / *The results is referred only to the sample analyzed. The present certificate of analysis cannot be reproduced even in part without permission of Responsible of certificate. This report is electronically valid, because it is controlled and exact copy of the signed original of the certificate of analysis, stored procedures according to requirements of Good Laboratory Practice.*

PROVA ANALITICA MICROBIOLOGICA	U.M	Intervallo di accettabilità
Carica microbica totale (ISO 21149:2009)	<10	Conforme (Limite: ≤10 CFU/g)
Staphylococcus aureus (Baird Parker Agar)	Assente	Conforme (Limite: Assente in 1 g)
Escherichia coli (VRBL Agar)	Assente	Conforme (Limite: Assente in 1 g)
Pseudomonas aeruginosa (Cetrimide Agar)	Assente	Conforme (Limite: Assente in 1 g)
Candida albicans (SDA+CF Agar)	Assente	Conforme (Limite: Assente in 1 g)
Aspergillus species (SDA+CF Agar)	Assente	Conforme (Limite: Assente in 1 g)

CFU/g = unità formanti colonia relative a 1 grammo di campione /
Colony Forming Units per 1 g of the sample.
U.M = Unità di Misura

***Riferimenti normativi/** *Normative references*
In base ai criteri di accettabilità in base alla 10^a edizione della Farmacopea Europea (PhEur), aggiornamento 07/2020, alla Linea guida *Scientific Committee on Consumer Safety* (SCCS_s_004 *Guidance for testing cosmetics*) il limite quantitativo della carica aerobica mesofila totale è stato indicato come corrispondente a ≤10 cfu/g e patogeni assenti.

Caratteristiche microbiologiche	Non è possibile la presenza o l'eventuale crescita di microrganismi, in quanto la presenza di ozonidi (derivati dall'ozonizzazione) determina caratteristiche antimicrobiche.

Data / *Date:* 16/07/2018
Revisione: 27/09/2021

UNIFE

(Responsabile scientifico
Firma / *Signature* Prof Pier Giorgio Balboni)
in collaborazione con Prof. P. G. Balboni
docente incaricato di insegnamento (MED/07)
Università degli studi di Ferrara

I risultati analitici si intendono riferiti esclusivamente al campione analizzato. Il presente Documento non può essere riprodotto neppure in forma parziale salvo approvazione scritta da parte del Responsabile. Questo report è valido elettronicamente, perché costituisce copia esatta controllata e firmata del certificato di analisi originale, conservato in accordo alle procedure di Norme di Buona Prassi di Laboratorio. / *The results is referred only to the sample analyzed. The present certificate of analysis cannot be reproduced even in part without permission of Responsible of certificate. This report is electronically valid, because it is controlled and exact copy of the signed original of the certificate of analysis, stored procedures according to requirements of Good Laboratory Practice.*

Reproduced with permission from the vendor
