# Supplementary material for: Physico-Chemical Investigation and Antimicrobial Efficacy of Ozonated Oils: The Case Study of Commercial Ozonated Olive and Sunflower Seed Refined Oils
Source: Molecules. 2024 Feb 1;29(3):679. doi: 10.3390/molecules29030679 (PMC10856119; doi:10.3390/molecules29030679)
Supplement: Supplementary file 1 [file molecules-29-00679-s001.zip › ozonated sunflower oil technical sheet.pdf]

CONSORZIO FUTURO IN RICERCA

in collaborazione con Prof. P. G. Balboni
docente incaricato di insegnamento (MED/07) *come cultore della materia "Microbiologia" - Sezione di Microbiologia Università degli studi di Ferrara*

SCHEDA TECNICA

**Committente / *Customer*:**<br><br>**OS s.r.l.**<br>Viale Fratelli Rosselli, 46 – 61121 PESARO (PU) - Italia	Data ricevimento /<br>*Date of the sample:* 16/09/2021<br><br>*Data inizio analisi /*<br>*Start date analisys:* 16/09/2021<br><br>*Data fine analisi /*<br>*End date analisys:* 24/09/2021<br>Revisione: 27/09/2021
**CODICE analisi / *Analisys CODE N°:*** 210921	
**Prodotto / Sample description**	
**PURE SUN 100% ozonized sunflower oil**	**LOTTO N° 210726**

Nome prodotto	Ozonized Sunflower Seed Oil (Heliantus annuus (sunflower) seed oil with Ozone
INCI NAME/IUPAC	Ozonized Sunflower Seed Oil (Heliantus annuus (sunflower) seed oil with Ozone
Composizione	Olio di semi di girasole ozonizzato
CAS no. (ECHA Europa)	-
EC no. (ECHA Europa)	924-751-7
Descrizione	Olio di semi di girasole ad elevato carico di Ozono (numero perossidi ≥ 3000 meqO_2/kg - metodo d'analisi Isco3) Olio di semi di girasole con presenza di gruppi idroperossidici nell'acido grasso dei trigliceridi, per reazione di addizione negli acidi grassi insaturi a doppio legame dopo il trattamento con ozono.<br>Prodotto per uso esterno a base di olio di girasole sottoposto ad ozonizzazione.
Categoria	Oli naturali
Aspetto a 20°C	Liquido oleoso traslucido, leggermente opalescente
Colore	Giallo paglierino
Odore	Profumazione caratteristica
Solubilità	Non solubile in acqua, solubile in glicerina /paraffina liposolubile in oli e disperdibile in emulsioni.

PARAMETRI CHIMICO FISICI	RISULTATO	Intervallo di accettabilità
Torbidità colore (NTU/FTU)	<1	Conforme (limiti: 0 ÷ 20)
Acidità mg KOH/g	28	Conforme (limiti: <30 mg KOH/g)
Densità (g/cm^3) a 20°C	0,990	Conforme (limiti: 0.920 ÷ 1.000)
Viscosità (mPas) Gir.5 rpm 20 a 25°C	180,0	Conforme (limiti: 80 ÷ 250)
Numero di ossidazione (meqO_2/kg) (metodo ISCO3)	3520	Conforme (limiti: 3000 ÷ 3600)
Conservazione	Conservare in luogo fresco, asciutto e al buio; lontano da fonti di calore; accuratamente chiuso senza contatto con l'ambiente esterno.	

I risultati analitici si intendono riferiti esclusivamente al campione analizzato. Il presente Documento non può essere riprodotto neppure in forma parziale salvo approvazione scritta da parte del Responsabile. Questo report è valido elettronicamente, perché costituisce copia esatta controllata e firmata del certificato di analisi originale, conservato in accordo alle procedure di Norme di Buona Prassi di Laboratorio. / *The results is referred only to the sample analyzed. The present certificate of analysis cannot be reproduced even in part without permission of Responsible of certificate. This report is electronically valid, because it is controlled and exact copy of the signed original of the certificate of analysis, stored procedures according to requirements of Good Laboratory Practice.*

**PARAMETRI MICROBIOLOGICI**	**U.M**	**Intervallo di accettabilità**
Carica microbica totale (ISO 21149:2009)	<10	Conforme (Limite: ≤10 CFU/g)
Staphylococcus aureus (Baird Parker Agar)	Assente	Conforme (Limite: Assente in 10 g)
Escherichia coli (VRBL Agar)	Assente	Conforme (Limite: Assente in 1 g)
Pseudomonas aeruginosa (Cetrimide Agar)	Assente	Conforme (Limite: Assente in 1 g)
Candida albicans (SDA+CF Agar)	Assente	Conforme (Limite: Assente in 1 g)
Aspergillus species (SDA+CF Agar)	Assente	Conforme (Limite: Assente in 1 g)

U.M. = Unità di misura
CFU/g = unità formanti colonia relative a 1 grammo di campione /
Colony Forming Units per 1 g of the sample.

Caratteristiche microbiologiche	Non è possibile la presenza o l'eventuale crescita di microrganismi, in quanto la presenza di ozonidi (derivati dall'ozonizzazione) determina caratteristiche antimicrobiche.

Settori di utilizzo	Cosmetico, farmaceutico come medical device ad uso topico, veterinario.
Concentrazione di utilizzo	Da 0,1% al 100% (in funzione della formulazione)

Data / *Date:* 24/09/2021

UNIFE

(Responsabile scientifico
Firma / *Signature* Prof Pier Giorgio Balboni)
in collaborazione con Prof. P. G. Balboni
docente incaricato di insegnamento (MED/07)
Università degli studi di Ferrara

I risultati analitici si intendono riferiti esclusivamente al campione analizzato. Il presente Documento non può essere riprodotto neppure in forma parziale salvo approvazione scritta da parte del Responsabile. Questo report è valido elettronicamente, perché costituisce copia esatta controllata e firmata del certificato di analisi originale, conservato in accordo alle procedure di Norme di Buona Prassi di Laboratorio. / *The results is referred only to the sample analyzed. The present certificate of analysis cannot be reproduced even in part without permission of Responsible of certificate. This report is electronically valid, because it is controlled and exact copy of the signed original of the certificate of analysis, stored procedures according to requirements of Good Laboratory Practice.*

Reproduced with permission from the vendor
